# Supplementary material for: OpenMM 7: Rapid development of high performance algorithms for molecular dynamics
Source: PLoS Comput Biol. 2017 Jul 26;13(7):e1005659. doi: 10.1371/journal.pcbi.1005659 (PMC5549999; doi:10.1371/journal.pcbi.1005659)
Supplement: S1 Source Code — (ZIP) [file pcbi.1005659.s002.zip › OpenMM-7.0.1-Source/docs/api-python/generated/simtk.openmm.openmm.RPMDMonteCarloBarostat.html]

RPMDMonteCarloBarostat — OpenMM 7.0.1.dev-5e86c4f documentation


# RPMDMonteCarloBarostat¶

*class* `simtk.openmm.openmm.``RPMDMonteCarloBarostat`(*\*args*)¶
:   This class is very similar to MonteCarloBarostat, but it is specifically designed for use with RPMDIntegrator. For each trial move, it scales all copies of the system by the same amount, then accepts or rejects the move based on the change to the total energy of the ring polymer (as returned by the integrator’s getTotalEnergy() method).

    `__init__`(*self*, *defaultPressure*, *frequency=25*) → RPMDMonteCarloBarostat¶
    :   \_\_init\_\_(self, defaultPressure) -> RPMDMonteCarloBarostat
        \_\_init\_\_(self, other) -> RPMDMonteCarloBarostat

        Create a MonteCarloBarostat.

        |  |  |
        | --- | --- |
        | Parameters: | - **defaultPressure** (*double*) – the default pressure acting on the system (in bar) - **frequency** (*int*) – the frequency at which Monte Carlo pressure changes should be attempted (in time steps) |

    Methods

    |  |  |
    | --- | --- |
    | `Pressure`(() -> std::string const &) | This is the name of the parameter which stores the current pressure acting on the system (in bar). |
    | `__init__`((self, defaultPressure[, frequency]) | \_\_init\_\_(self, defaultPressure) -> RPMDMonteCarloBarostat |
    | `getDefaultPressure`((self) -> double) | Get the default pressure acting on the system (in bar). |
    | `getForceGroup`((self) -> int) | Get the force group this Force belongs to. |
    | `getFrequency`((self) -> int) | Get the frequency (in time steps) at which Monte Carlo pressure changes should be attempted. |
    | `getRandomNumberSeed`((self) -> int) | Get the random number seed. |
    | `setDefaultPressure`(self, pressure) | Set the default pressure acting on the system. |
    | `setForceGroup`(self, group) | Set the force group this Force belongs to. |
    | `setFrequency`(self, freq) | Set the frequency (in time steps) at which Monte Carlo pressure changes should be attempted. |
    | `setRandomNumberSeed`(self, seed) | Set the random number seed. |
    | `usesPeriodicBoundaryConditions`((self) -> bool) | Returns whether or not this force makes use of periodic boundary conditions. |

    *static* `Pressure`() → std::string const &¶
    :   This is the name of the parameter which stores the current pressure acting on the system (in bar).

    `getDefaultPressure`(*self*) → double¶
    :   Get the default pressure acting on the system (in bar).

        |  |  |
        | --- | --- |
        | Returns: | the default pressure acting on the system, measured in bar. |
        | Return type: | double |

    `setDefaultPressure`(*self*, *pressure*)¶
    :   Set the default pressure acting on the system. This will affect any new Contexts you create, but not ones that already exist.

        |  |  |
        | --- | --- |
        | Parameters: | **pressure** (*double*) – the default pressure acting on the system, measured in bar. |

    `getFrequency`(*self*) → int¶
    :   Get the frequency (in time steps) at which Monte Carlo pressure changes should be attempted. If this is set to 0, the barostat is disabled.

    `setFrequency`(*self*, *freq*)¶
    :   Set the frequency (in time steps) at which Monte Carlo pressure changes should be attempted. If this is set to 0, the barostat is disabled.

    `getRandomNumberSeed`(*self*) → int¶
    :   Get the random number seed. See setRandomNumberSeed() for details.

    `setRandomNumberSeed`(*self*, *seed*)¶
    :   Set the random number seed. It is guaranteed that if two simulations are run with different random number seeds, the sequence of Monte Carlo steps will be different. On the other hand, no guarantees are made about the behavior of simulations that use the same seed. In particular, Platforms are permitted to use non-deterministic algorithms which produce different results on successive runs, even if those runs were initialized identically.

        If seed is set to 0 (which is the default value assigned), a unique seed is chosen when a Context is created from this Force. This is done to ensure that each Context receives unique random seeds without you needing to set them explicitly.

    `usesPeriodicBoundaryConditions`(*self*) → bool¶
    :   Returns whether or not this force makes use of periodic boundary conditions.

        |  |  |
        | --- | --- |
        | Returns: | true if force uses PBC and false otherwise |
        | Return type: | bool |

    `__delattr__`¶
    :   x.\_\_delattr\_\_(‘name’) <==> del x.name

    `__format__`()¶
    :   default object formatter

    `__getattribute__`¶
    :   x.\_\_getattribute\_\_(‘name’) <==> x.name

    `__hash__`¶

    `__reduce__`()¶
    :   helper for pickle

    `__reduce_ex__`()¶
    :   helper for pickle

    `__sizeof__`() → int¶
    :   size of object in memory, in bytes

    `__str__`¶

    `getForceGroup`(*self*) → int¶
    :   Get the force group this Force belongs to.

    `setForceGroup`(*self*, *group*)¶
    :   Set the force group this Force belongs to.

        |  |  |
        | --- | --- |
        | Parameters: | **group** (*int*) – the group index. Legal values are between 0 and 31 (inclusive). |

High performance molecular simulation on GPUs


### Navigation

- Application Layer
- Library Layer
  - Core Objects
  - Forces
  - Integrators
  - Extras

©2015, Stanford University and the Authors.
|
Powered by Sphinx 1.3.1
& Alabaster 0.7.7
|
Page source
